# Effect of Sugarcane Burning or Green Harvest Methods on the Brazilian Cerrado Soil Bacterial Community Structure

**DOI:** 10.1371/journal.pone.0059342

**Published:** 2013-03-22

**Authors:** Caio T. C. C. Rachid, Adriana L. Santos, Marisa C. Piccolo, Fabiano C. Balieiro, Heitor L. C. Coutinho, Raquel S. Peixoto, James M. Tiedje, Alexandre S. Rosado

**Affiliations:** 1 Center for Nuclear Energy in Agriculture, Universidade de São Paulo, Piracicaba, São Paulo, Brazil; 2 Institute of Microbiology Paulo de Góes, Federal University of Rio de Janeiro, Rio de Janeiro, Rio de Janeiro, Brazil; 3 Embrapa Solos, Rua Jardim Botânico, Rio de Janeiro, Rio de Janeiro, Brazil; 4 Center for Microbial Ecology, Michigan State University, East Lansing, Michigan, United States of America; Graz University of Technology (TU Graz), Austria

## Abstract

**Background:**

The Brazilian Cerrado is one of the most important biodiversity reservoirs in the world. The sugarcane cultivation is expanding in this biome and necessitates the study of how it may impact the soil properties of the Cerrado. There is a lack of information especially about the impacts of different sugarcane management on the native bacterial communities of Cerrado soil. Therefore, our objective was to evaluate and compare the soil bacterial community structure of the Cerrado vegetation with two sugarcane systems.

**Methods:**

We evaluated samples under native vegetation and the impact of the two most commonly used management strategies for sugarcane cultivation (burnt cane and green cane) on this diversity using pyrosequencing and quantitative PCR of the rrs gene (16S rRNA).

**Results and Conclusions:**

Nineteen different phyla were identified, with Acidobacteria (≈35%), Proteobacteria (≈24%) and Actinobacteria (≈21%) being the most abundant. Many of the sequences were represented by few operational taxonomic units (OTUs, 3% of dissimilarity), which were found in all treatments. In contrast, there were very strong patterns of local selection, with many OTUs occurring only in one sample. Our results reveal a complex bacterial diversity, with a large fraction of microorganisms not yet described, reinforcing the importance of this biome. As possible sign of threat, the qPCR detected a reduction of the bacterial population in agricultural soils compared with native Cerrado soil communities. We conclude that sugarcane cultivation promoted significant structural changes in the soil bacterial community, with Firmicutes phylum and Acidobacteria classes being the groups most affected.

## Introduction

Brazilian Cerrado (neotropical savanna vegetation) covers more than 20% of the country’s surface and is one of the most diverse biomes (major biotic community) in the world. In addition, this biome is classified as a high-priority area for biodiversity conservation [1]–[3].

However, around 40% of the Cerrado land has been converted for agricultural and livestock production, a process that has been very active during the last two decades [3], [4]. Agricultural expansion has been stimulated by incentives from the Brazilian Federal government, e.g. the Cerrado Development Program, the Cattle-Raising Development Council and the Alcohol Program and multilateral agencies such as the Nippo-Brazilian Cooperation Program for the Development of the Cerrado [5]. This scenario has resulted in the Cerrado region producing 49% of the grain, 41% of the milk and 40% of the beef in Brazil [6].

Recently, the expansion of sugarcane production in the Cerrado has received renewed attention because sugarcane is an important material for agroenergy and bioethanol production [7]. The cultivated areas in the states of Goiás and Mato Grosso do Sul have grown more than 300% in the last 5 years [8]. This expansion is the outcome of the Brazilian biofuel program and occurs mainly through the replacement of established agricultural areas (pastures and soy and corn fields) by new sugarcane areas [9], [10].

The impact of sugarcane on different components of the agroecosystem (water, soil structure, greenhouse emissions, enzyme activities, soil carbon stocks and fertility) is well documented, although mainly for the Brazilian Atlantic Forest biome [11]–[16]. Briefly, these works show that residue management (the use of burnt cane techniques, in which, pre-harvest burning accompanies manual harvest, vs. green sugarcane, in which fire is not used and which is associated with mechanical harvest) has little effect on C stocks in the short term (2–4 years). However, these studies have shown that labile organic matter fractions, such as microbial biomass, particulate organic matter and soil enzyme activity, and some physical measures, such as the mean weight diameter of water-stable soil aggregates, are more sensitive to these soil management methods.

Land use change can significantly influence the bacterial community structure and abundance, which, in turn, is correlated with soil biogeochemical processes and ecosystem productivity [17]–[20]. Therefore, understanding the microbial community structure and its relationship with the changes in land use and management are fundamental to understanding ecosystems.

There is a lack of information about the bacterial diversity of the Cerrado soil, and the impacts of different management strategies on native bacterial communities, or even more comprehensives studies, such as clone library screening [21], [22], to document bacterial community structure. Next-generation sequencing technology such as 454 pyrosequencing provides much greater capacity to improve the knowledge about the soil microbial diversity of this biome. Only one recent work used this technology to study the Cerrado bacterial diversity, however, without evaluating the impact of agriculture on soil microbiology [23].

Our goal was to evaluate the bacterial community structure of Cerrado soil samples under native vegetation and the impact of the two most commonly used management practices of sugarcane cultivation, burnt cane and green cane, on this community using pyrosequencing and quantitative real-time PCR.

## Materials and Methods

### Field Site and Sampling

The study area (17° 55′ 35′′ S, 50° 08′ 36′′ W) was located at the municipality of Porteirão, state of Goiás, Brazil. The regiońs climate is classified as Aw (Köppen), with annual average rainfalls exceeding 1500 mm year^−1^ and annual average air temperatures of 23.1°C. The soil is a eutrophic Latossolo vermelho (Ferralsols), which is characterised by high levels of base saturation (>50%). Although the area is flat, petroplinthites (lateritic nodules or concretions) are found in the subsurface, which may restrict drainage and exhibit concretionary characteristics (Oliveira et al. 1992).

The field had been previously used for cotton, soybean and sunflower production, and was converted to sugarcane cultivation in 2002. The samples were collected in September 2008, during the sugarcane growth stage, approximately 7 to 8 months after bud germination (after six yearly harvest cycles). The field was divided into three regions (split-plot) in which three different regimes were applied:

- Burnt sugarcane – Before harvest, the sugarcane was ignited to remove the leaves. The stem was then manually harvested. After harvest, the soil remained uncovered (17°55′32.05′′S 50° 8′50.64′′W).
- Green sugarcane – Harvest was performed using a machine that separates the sugarcane leaves from the stems. The leaves are then returned to the soil. After harvest, the soil remained covered by the vegetal residues (17°55′35.83′′S 50° 8′41.10′′W).
- Cerrado – covered by a dense formation of trees up to 4 metres tall, as a typical Cerradão formation [24]. It represents the soil in a more natural condition, and received no addition of fertilizers (17°55′32.52′′S 50° 8′37.92′′W).

The sugarcane treatments had 6 years of application before the sampling. The sizes of the burnt sugarcane, green sugarcane and Cerrado areas were 23.5, 9.9 and 2.9 ha, respectively. The three treatments were very close to one another, less than 300 m apart. To allow replication, per treatment, three 5×5 m subplots were defined randomly (approximately 10 m of distance from each other). The soil was collected, five points per subplot (which were pooled) approximately to 10 cm depth, using a core borer. All soil was collected in the same day (approximately 2.5 kg), mixed and transported to the operational base in normal temperature within 2 hours. Then, smaller amounts of the soil (10 g) were separated, stored in centrifuge tubes and frozen. The soil was kept frozen until DNA extraction. This present study is the continuation of a previous work, which used the same soil samples for physicochemical and biological analysis. Information about the physical and chemical properties of the sites has been previously described [25], and the general soil characterization can be found in the supplementary material (Table S1).

### Real-time PCR Analysis of the rrs Gene

Quantitative PCR was performed on the ABI PRISM® SDS 7000 (PE Applied Biosystems). Amplification reactions were performed with the SYBR Green PCR master mix (Applied Biosystems) using the primers 357F (5′ CTA CGG GRS GCA G 3′) and 529R (5′ CGC GGC TGC TGG CAG 3′) [26] specific for bacteria, at a concentration of 300 nM each, and a DNA template volume of 1 µl (≈ 20 ng) was added to 24 µl of PCR master mix in MicroAmp Optical 96-well reaction plates. The real-time PCR thermocycling steps for all primer sets were as follows: 50°C for 2 min; 95°C for 10 min; and 40 cycles of 95°C for 1 min, 50°C for 1 min and 60°C for 1 min. In all experiments, appropriate negative controls containing no template DNA were subjected to the same procedure to exclude or detect any possible contamination or carryover. Melting curves were also routinely checked to confirm the purity of the amplified products.

Standard curves were obtained by plotting Ct (threshold cycle) as a function of the log of the copy number of the target DNA. Tenfold serial dilutions of the plasmids containing the standard sequences, ranging from 10^1^ to 10^8^ serial dilutions, were used as the target DNA. All measurements were performed in triplicate per sample. To test the difference among the treatments, the data we performed one anova with log transformed data.

### Bacterial Pyrosequencing and Sequence Processing

Soil (0.5 g) DNA was extracted from triplicate samples using the FastDNA® Spin Kit for Soil and the FastPrep® equipment (Bio 101, CA, USA) according to the manufacturer’s instructions. The extracted DNA was submitted to PCR amplification, targeting hyper-variable region 4 of the rrs gene (16S rRNA) using the primers 563F and 802R [27]. The reactions were performed as described by [28]. Equimolar amounts of the PCR products were submitted to pyrosequencing on a Genome Sequencer FLX system (454 Life Sciences, USA) at Michigan State University. The sequences are available at the NCBI Sequence Read Archive under the following accession numbers: SRR077401.2, SRR077402.2, SRR077403.2, SRR077404.2, SRR077405.2, SRR077406.2, SRR077407.2, SRR077408.2, and SRR077409.2.

The raw sequences were processed by using Mothur v. 1.26.0. [29]. The flowgrams were submitted to Pyronoise [30] to reduce error in the retained data set. Additionally all sequences missing the forward primer and/or had a length smaller than 190 bases were removed. The high quality sequences were then aligned using Silva reference database and the chimeras were detected and eliminated. The overall quality processing removed around 3,600 sequences, and resultant alignments file with only high quality sequences served as inputs to construct the distance matrix and to cluster the sequences into OTUs. To avoid the bias of analysing data with different sequences numbers, we performed all the analyses (with exception of the taxonomic assignment for relative abundance) with a normalised number of sequences for all treatments. The clusters were constructed at a 3% dissimilarity and were used to generate predictive rarefaction models and make calculations using the richness indices Ace and Chao1 [31] and Shannon’s diversity index [32]. Mothur was also used to perform the NMDS ordination of the samples and to test the significance of the differences among the treatments based on the Weight Fast UniFrac and Amova tests.

To obtain the taxonomic assignment and relative abundance of the different bacterial groups the sequences were submitted to RDP-II Classifier using an 80% confidence threshold [33], [34].

## Results and Discussion

A total of 22,424 high-quality sequences were obtained from all samples. The minimum and maximum number of sequences for each sample ranged from 1322 (GC2) to 5164 (GC1), respectively. When analysed together, the treatments showed more than 3000 different operational taxonomic units (3% dissimilarity). The number of OTUs was similar in all analysed samples, with no significant differences among the treatments (Table 1). The most abundant OTUs were composed mainly by uncultured bacteria from different phyla. The summary of them can be found in the supplementary material (Table S2). There were also no differences (p>0.05) for the Chao1, Ace and Shannon diversity estimators among the treatments (Table 1), nor in the rarefaction curve (Figure S1). Although the pyrosequencing did not detect a significant influence in the bacterial diversity index cited above, the qPCR did reveal a significant decrease in the number of rrs gene (16S rRNA) copies in the cultivated sites, GC ≈ 3.3 10^8^±6.10^7^ (average and standard deviation, per gram of dry soil) and BC ≈ 2.2 10^8^±8.10^7^, compared with the natural area CE ≈ 6.3 10^8^, ±6.10^7^ suggesting an overall suppression of the bacterial community in the cultivated areas.

**Table 1:** Estimated OTU richness and diversity index for rrs gene of Cerrado soil samples under native vegetation and sugarcane cultivation.

Treatments	Sequence number*	OTUs	Estimated OTU richness		Shannon
			CHAO1	ACE	
CE1	1306	595	1422(1216;1698)	2433(2197;2704)	5.85(5.78;5.92)
CE2	1306	639	1764(1486;2135)	3082(2800;3401)	5.99(5.92;6.05)
CE3	1306	596	1296(1122;1527)	2062(1863;2292)	5.88(5.81;5.95)
GC1	1306	613	1415(1217;1678)	2286(2071;2534)	5.95(5.89;6.02)
GC2	1306	578	1300(1112;1554)	1868(1696;2066)	5.91(5.85;5.97)
GC3	1306	624	1530(1305;1830)	2247(2033;2494)	5.99(5.93;6.06)
BC1	1306	705	2033(1721;2440)	3840(3496;4226)	6.11(6.04;6.17)
BC2	1306	565	1228(1054;1463)	1684(1527;1867)	5.87(5.80;5.93)
BC3	1306	598	1556(1312;1882)	2644(2394;2928)	5.88(5.81;5.95)
Total	11754				

* Normalized number of sequences.

Inside brackets, the lower and upper limits of values between the 95% confidence intervals).

With respect to the phylogenetic composition of the samples, many of the sequences from all treatments (from 32% to 43%) could only be assigned to the Bacteria domain and had no classification at the phylum level. This percentage of unclassified bacteria is higher than that already described in other environments such as grassland, pasture, and agricultural systems in Texas (5% to 10% unclassified) [35]; German forest and grassland soils (10 to 25%) [36]; oak forest soil (18 to 22%) [37] and Amazonian dark earth (26 to 36%) [38]. The high fraction of unclassified sequences may have two possible explanations: first, the sequence size may be too short for accurate classification using the bootstrap cut-off value of 80%, and second, the database may not be complete enough to include all different components within each phylum and, as a result, be missing some comparative elements with which to classify all microbial diversity. The second reason is more plausible to explain the high level of unclassified sequences because the sequences length were larger than 200 bp, which is enough to classify known bacteria at the phylum level with 99.5% accuracy using the Classifier tool [34]. Furthermore, the high level of unclassified bacteria emphasises the high degree of undiscovered biodiversity found in the “hotspot” Cerrado biome.

**Figure 1:**
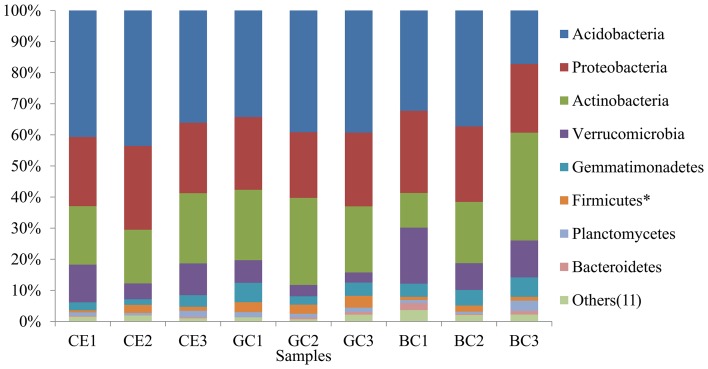
The relative frequencies of the different phyla found in Cerrado (CE), Green Cane (GC) and Burnt Cane (BC) samples.

Among the classified sequences, the most abundant phyla found in all treatments were (in rank order): Acidobacteria, Proteobacteria, Actinobacteria, Verrucomicrobia, Gemmatimonadetes, Firmicutes, Planctomycetes and Bacteroidetes (Figure 1). Eleven other phyla were found in at least one sample (Chloroflexi, TM7, Armatimonadetes, Chlamydiae, WS3, OD1, Nitrospira, Cyanobacteria/Chloroplast, Spirochaetes, BRC1 and Fusobacteria). These data are in accordance with other studies that used pyrosequencing or clone libraries which also found these phyla as the most abundant in soils [21], [23], [37], [39]–[41].

However, the predominance of Acidobacteria compared with the other phyla has not been observed in most other studies. In general, Proteobacteria have the highest frequency in previous studies [21], [37], [39]–[41], with exception of another Cerrado soil survey that found Acidobacteria as the most dominant group [23], highlighting a pattern for this biome. Moreover, the relative frequency of Acidobacteria has been shown to range from 2.4% to 78.5% in different soils, varying mainly due to differences in pH, with Acidobacteria more abundant in acid soils, but also in correlation with other soil properties [42].

Our data suggest that the role of the phylum Bacteroidetes is of minor relevance in our area because it accounts for only a very small fraction of the entire bacterial diversity (0.1 to 3% of the sequences), in contrast with other studies in which this phylum is very abundant and accounts for up to 20% of the classified sequences [39], [40]. The phylum Cyanobacteria, represented by five sequences, was only found in the BC treatment in the present study.

Among the most abundant phyla, only Firmicute sequences had significantly different distributions among the treatments (p<0.01), with a higher frequency in the GC treatment than in the others. Despite not being significant, the relative frequency of Acidobacteria decreased in the cultivated areas, mainly in the burnt cane plots, as can be noted by comparing the average values of the frequency of this phylum in CE (41%) to those in GC (38%) and BC (29%). However, the average relative frequency of Verrucomicrobia tended to be higher in the BC area (13%) than in CE (9.3%) and GC (5%).

**Figure 2:**
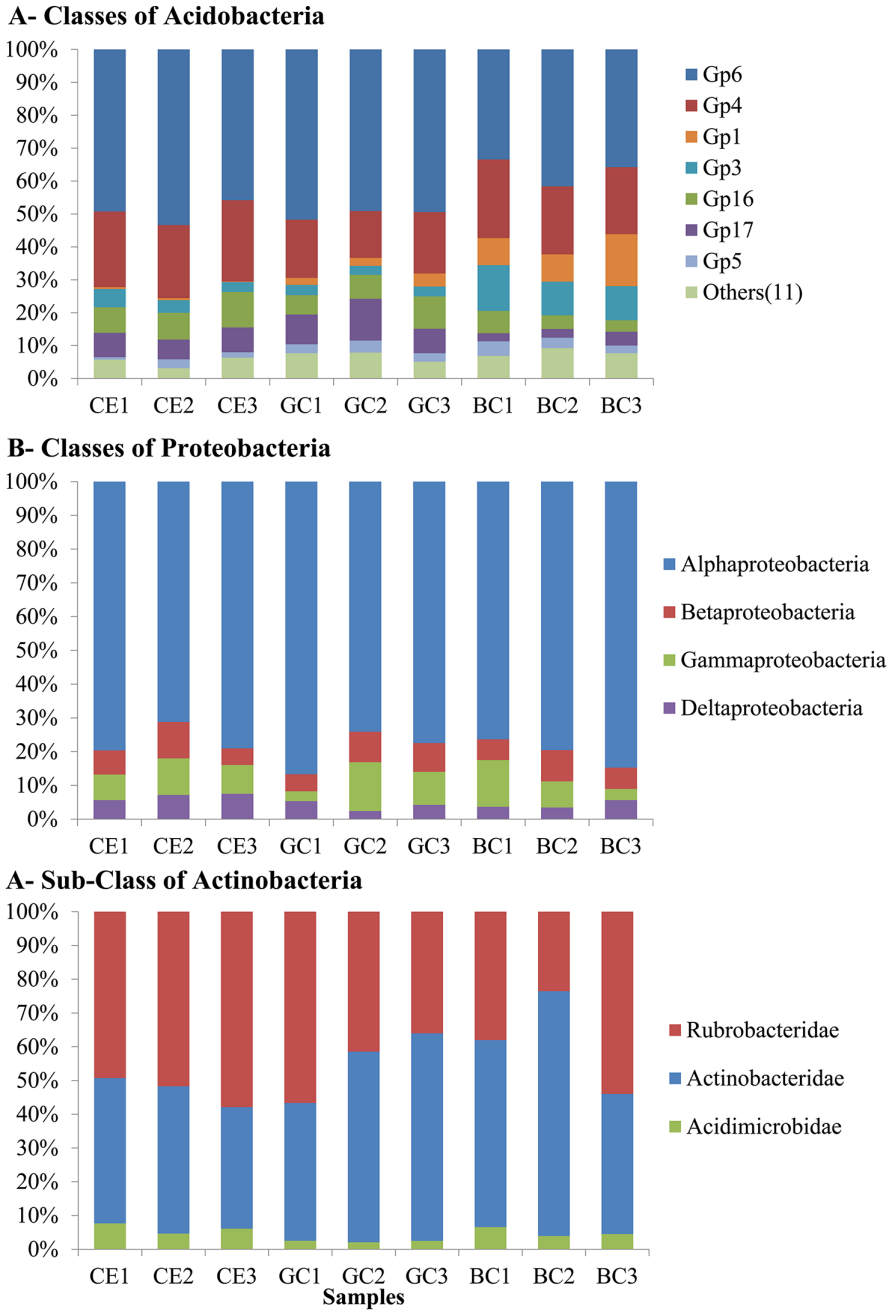
The relative frequencies of the different classes found in Cerrado (CE), Green Cane (GC) and Burnt Cane (BC) samples for the following phyla: A – Acidobacteria, B – Proteobacteria, C – Actinobacteria.

The Acidobacteria phylum was represented by eight different classes (Figure 2A), which are, in the order of decreasing abundance, Gp6> Gp4> Gp1> Gp3> Gp7> Gp2> Gp22 and Gp25. The highest difference among the treatments was found in these classes. The burnt cane responded with a decrease in the relative frequency of Gp6 (p<0.001) and with an increase in the relative abundances of Gp1 (p<0.001) and Gp3 (p<0.001). These alterations can be explained by small variations in the soil pH. Jones and colleagues [42] showed that Gp6 correlated positively with pH, whereas Gp1 and Gp3 correlated negatively. The burnt cane exhibited a slight soil acidification, pH 5.8±0.2 (average and standard deviation) compared with the green cane and Cerrado treatments, ph 6.6±0.08 and 6.4±0.06 respectively, and this change in the pH was correlated with the variation in the relative frequencies of these groups. Additionally, some other differences were observed, most specifically with the class Gp4 being significantly different between CE and GC and the class Gp25 being different between CG and BC.

The Proteobacteria phylum (Figure 2B) was fully dominated by the Alphaproteobacteria class, accounting for 70% (CE2) to 84% (GC1) of the relative frequencies, whereas Betaproteobacteria, Gammaproteobacteria and Deltaproteobacteria were also found but in low frequencies. Only Deltaproteobacteria differed significantly, being higher in the CE (p = 0.01) than in the other treatment areas, revealing a higher sensitivity to cropping. These data differ from those found by Roesch and colleagues [39], who studied different agricultural soils in North America (including a sugarcane field) and Brazil using pyrosequencing. These authors found that Betaproteobacteria was the most abundant class of Proteobacteria in all places except in Brazil, where Gammaproteobacteria was more frequent. However, other studies found a predominance of Alphaproteobacteria followed by Betaproteobacteria [36], [37].

The third most abundant phylum, Actinobacteria, was composed basically by the sub-classes Rubrobacteridae and Actinobacteridae, but Acidimicrobidae was also found at a low frequency (Figure 2C). This finding is in accordance with that of Janssen [41], who described the presence of these three sub-classes in some clone libraries, with a higher average frequency of the former two classes than of Acidimicrobidae.

Only Bacilli and Clostridia comprise the Firmicute phylum, with 70% to 100% of the sequences belonging to the Bacilli class (Figure S2A). Using pyrosequencing, Teixeira et al. [28] showed that Firmicute was an important phylum in rhizospheric Antarctic soil, but the major component (approximately 80%) of this phylum in this environment was Clostridia. However, Janssen (2006) in a review of clone libraries showed that Bacilli are more common than Clostridia in the majority of soils.

Finally, Verrucomicrobia was composed mainly of the Spartobacteria class in all treatments, with a Subdivision 3 class and a few sequences of Opitutae and Verrucomicrobiae occurring (Figure S2B).

**Figure 3:**
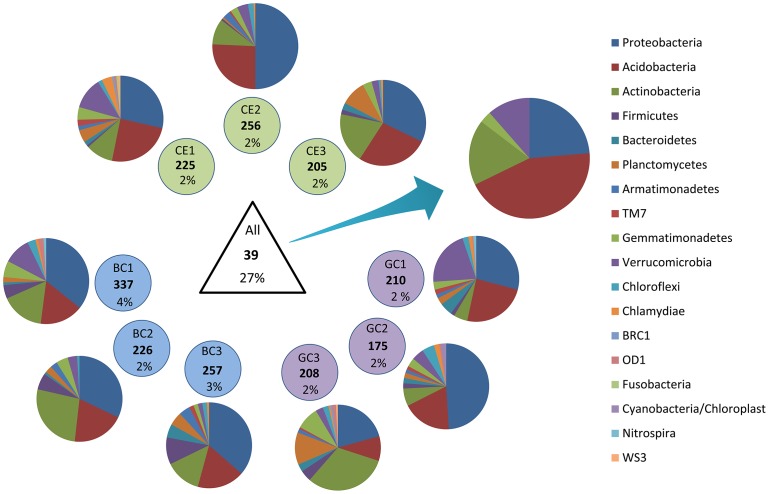
The general distribution of different OTUs among and within the samples and the relative frequencies of the phyla found in each situation (only the classified sequences). The written information represents the code of the sample, the number of OTUs found and the proportion of the sequences that these OTUs represent. Each written circle shows the data for the OTUs found only in that sample. The triangle shows the data for the OTUs found in all samples.

To understand the distribution of OTUs among the different treatments, the sequences of all three samples from all treatments were clustered together. The results showed that only 39 out of the 3000 OTUs were shared by all samples (Figure 3), indicating that few species had a broad distribution. Despite the low number, these shared OTUs accounted for 27% of all sequences, revealing that these species were very dominant. However, there were many OTUs that occurred only in one sample (Figure 3), and the number of OTUs occurring exclusively in one sample ranged between 175 (GC2) to 337 (BC1). The number of exclusive OTUs was much higher than those shared between treatments. This result reveals a high level of local microniche selection. However, these OTUs were represented by a very small proportion of sequences, indicating that they were basically composed of rare organisms.

The taxonomic assignment of the OTUs shared by all samples reveals a contrasting frequency of phyla compared with those that occurred in only one sample. The OTUs with a broad distribution belonged to only five phyla and had a higher predominance of Acidobacteria, whereas the taxonomic assignment of the OTUs that occurred only in one sample revealed the presence of at least ten different phyla for each sample. The relative frequencies of the taxonomic assignment of those samples were also different. There was a predominance of Proteobacteria for all samples except BC3 and, interestingly, a high frequency of Planctomyces in many samples, especially in BC3 and CE3. Curiously, no sequences belonging to Planctomyces were found among the OTUs with a broad distribution.

**Figure 4:**
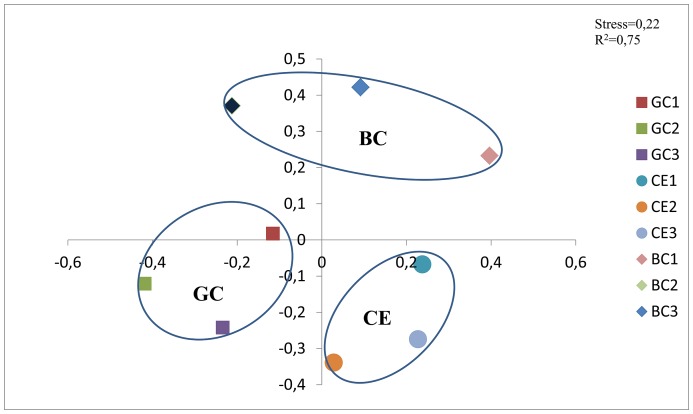
The NMDS ordination of the pyrosequencing data of Cerrado (CE), Green Cane (GC) and Burnt Cane (BC) samples.

The land management had a significant influence on the microbial community, as revealed by the non-metric multidimensional scaling (NMDS) analysis of the pyrosequencing data (Figure 4), despite the low variation in the relative abundance of the different phyla among the treatments. A clear clustering of each treatment in a distinct region of the plot could be detected. To test the significance of this clustering, the Weight Unifrac and Amova tests were used. Both tests showed significant differences when all treatments were analysed together (p = 0.001), or in pairwise comparisons. Therefore, the NMDS analysis suggests that the cultivation of sugarcane plays an important role in the transformation of the microbial community. Additionally, the microbial community selected for by green cane management seems to be more similar to the microbial community observed in areas under native vegetation compared than that selected by burnt cane, indicating a lower impact of green cane management on microbial bioindicators.

### Conclusion

Cerrado soil is a very complex ecosystem with a great bacterial diversity, of which a substantial fraction remains undiscovered. The pyrosequencing of Cerrado soils has revealed a high level of unclassified bacteria. Sugarcane cultivation reduced the bacterial population on ferralsols-sampled soil, with Acidobacteria classes being the most influenced by this land use. In all areas, up to nineteen phyla were identified, with Acidobacteria, Proteobacteria and Actinobacteria being the most abundant. Firmicute sequences exhibited significantly different distributions among the treatments (p<0.01), with a higher frequency in the GC treatment. In contrast, Deltaproteobacteria occurred at higher levels on CE (p = 0.01), revealing a higher sensitivity to cropping. Finally, significant structural changes of the community were observed, with the burnt cane management having a greater impact than green cane management on the native Cerrado soil communities. In this work, we demonstrated the impact in the microbial community resulting from the use of the Cerrado to cultivation of the sugarcane. However, due to the great variability of the Cerrado ecosystem, further research is required to confirm these findings, with soil samples from different sites and seasons, in order to address the impact due to changes in management over the years.

## Supporting Information

Figure S1 **A rarefaction curve of the Cerrado (CE), Green Cane (GC) and Burnt Cane (BC) samples, constructed with Mothur and using 3% of dissimilarity.**
(TIF)

Figure S2 **The relative frequencies of the different classes found in Cerrado (CE), Green Cane (GC) and Burnt Cane (BC) samples for the following phyla: A – Firmicutes, B – Verrucomicrobia.**
(TIF)

Table S1 **Average values of soil properties.**
(DOCX)

Table S2 **Closest relatives, and classification at phylum level of the most abundant OTUs (more than 1% of the sequences) from each sample.** Representative sequences of each OTU were selected using Mothur, and the Ribossomal Database Project Seqmatch tool was used to establish the closest match for each OTU.
(DOCX)
