# Supplementary figures and images for: Effect of Sugarcane Burning or Green Harvest Methods on the Brazilian Cerrado Soil Bacterial Community Structure
Source: PLoS One. 2013 Mar 22;8(3):e59342. doi: 10.1371/journal.pone.0059342 (PMC3606482; doi:10.1371/journal.pone.0059342)

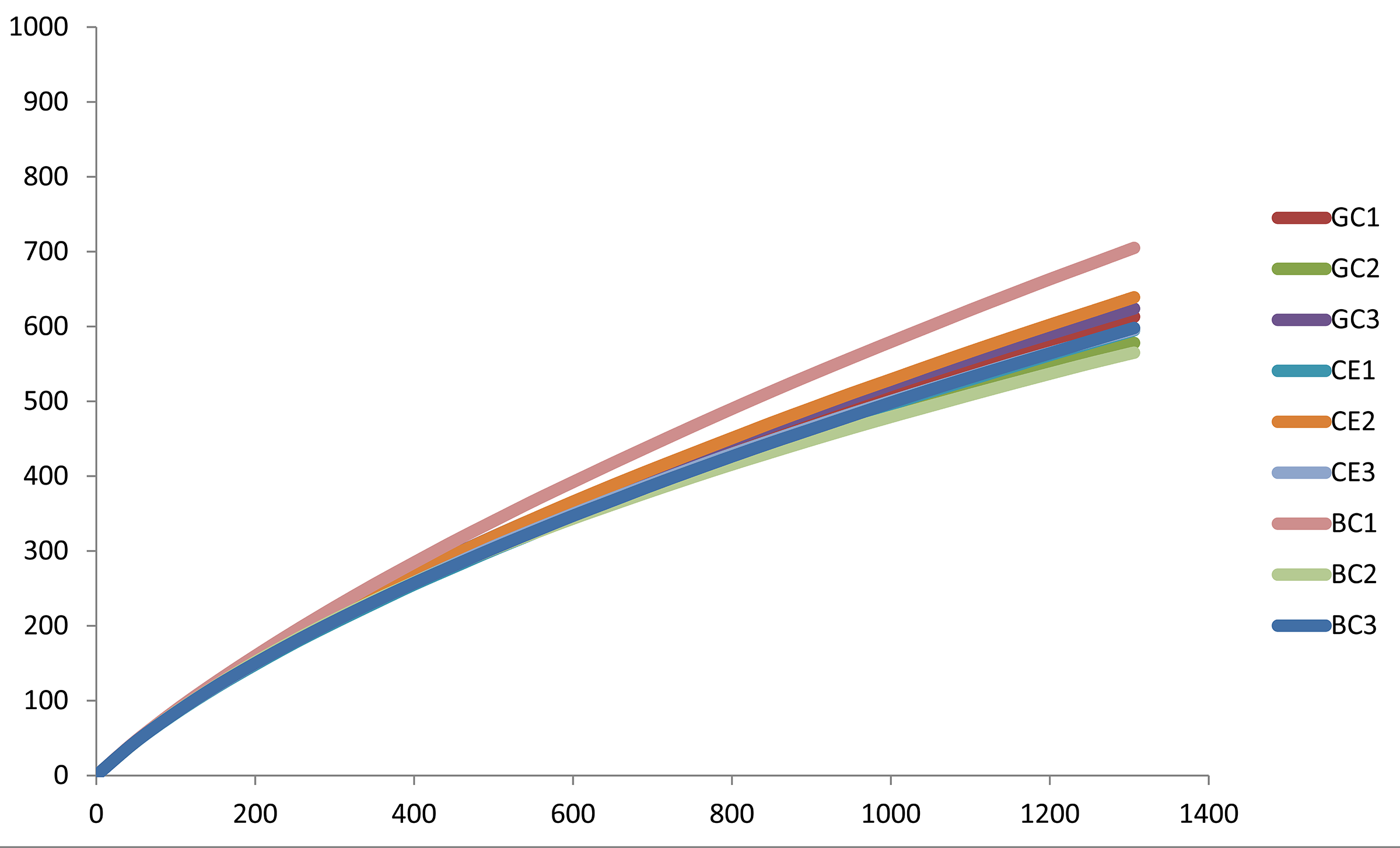

Supplement: Figure S1 — A rarefaction curve of the Cerrado (CE), Green Cane (GC) and Burnt Cane (BC) samples, constructed with Mothur and using 3% of dissimilarity. (TIF) [file pone.0059342.s001.tif]

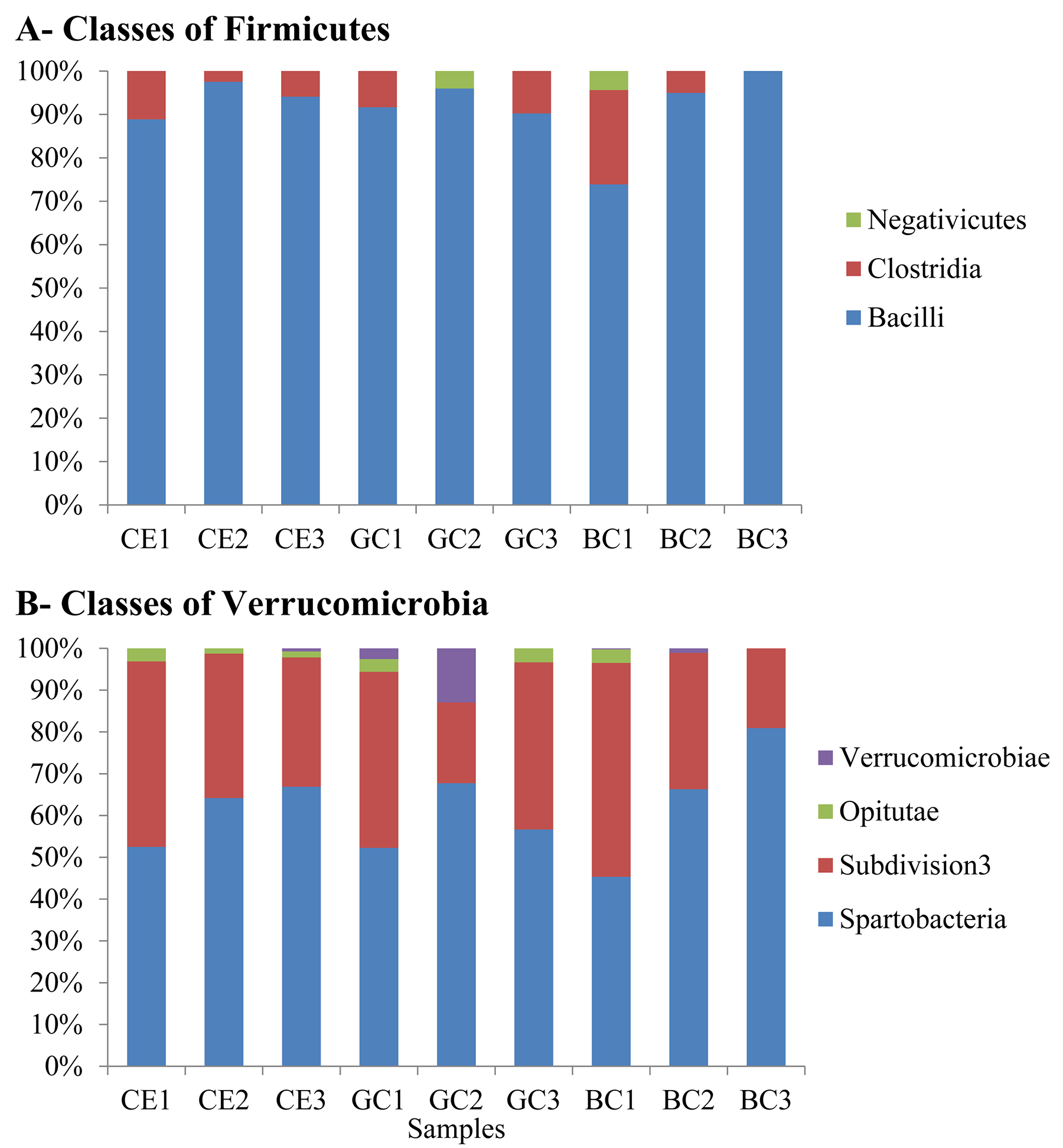

Supplement: Figure S2 — The relative frequencies of the different classes found in Cerrado (CE), Green Cane (GC) and Burnt Cane (BC) samples for the following phyla: A – Firmicutes, B – Verrucomicrobia. (TIF) [file pone.0059342.s002.tif]
