# Supplementary material for: Effect of Sugarcane Burning or Green Harvest Methods on the Brazilian Cerrado Soil Bacterial Community Structure
Source: PLoS One. 2013 Mar 22;8(3):e59342. doi: 10.1371/journal.pone.0059342 (PMC3606482; doi:10.1371/journal.pone.0059342)
Supplement: Table S1 — Average values of soil properties. (DOCX) [file pone.0059342.s003.docx]

**Table S1** –

| Parameters | Treatment | | |
| --- | --- | --- | --- |
|  | Control | Green cane | Burnt cane |
| pH | 6.6^a^ | 6.4^a^ | 5.8^b^ |
| Exchangeable Al | BD | BD | BD |
| Exchangeable Ca | 11.4^a^ | 10.^b^ | 4.3^c^ |
| Exchangeable Mg | 3.9^a^ | 2.1^b^ | 1.6^c^ |
|  |  |  |  |
| Exchangeable Na | 1.7^a^ | 2.8^a^ | BD |
| Exchangeable K | 306.6^b^ | 735.6^a^ | 280.0^b^ |
|  |  |  |  |
| Exchangeable H+Al | 4.8^b^ | 5.0^b^ | 6.5^a^ |
| Total P | 102.3^a^ | 34.6^ab^ | 32.6^b^ |
| SB^1^ | 16.1^a^ | 14.2^b^ | 6.6^c^ |
| CEC^2^ | 20.9^a^ | 19.0^b^ | 13.1^c^ |
| V^3^ | 77.0^a^ | 74.7^a^ | 50.4^b^ |
| Total C | 12.5^a^ | 6.7^b^ | 15.9^a^ |
| Total N | 0.70^a^ | 0.30^b^ | 0.90^a^ |
|  |  |  |  |
| C:N | 17.9^b^ | 22.3^a^ | 16.4^b^ |

The numbers represent average values (n=3). Averages followed by the same letter in each line are not statistically different (5%) from each other according to the Kolmogorov-Smirnov test for Ca, Mg, Na, K, P and V; and according Tukey test for the rest. BD - Below the detection limit of the technique. ^1^Sum of bases (sums of the Ca, Mg, Na and K content in cmol_c_ dm^-3^). ^2^Cation exchange capacity (sums of SB and H+Al). ^3^Percent base saturation (SB divided by CEC). Parameters units: Al, Ca, Mg, H+Al, P, SB, CEC (cmol_c_ dm^-3^), Na, K (mg dm^-3^), V (%).
