# Supplementary material for: Effect of Sugarcane Burning or Green Harvest Methods on the Brazilian Cerrado Soil Bacterial Community Structure
Source: PLoS One. 2013 Mar 22;8(3):e59342. doi: 10.1371/journal.pone.0059342 (PMC3606482; doi:10.1371/journal.pone.0059342)
Supplement: Table S2 — Closest relatives, and classification at phylum level of the most abundant OTUs (more than 1% of the sequences) from each sample. Representative sequences of each OTU were selected using Mothur, and the Ribossomal Database Project Seqmatch tool was used to establish the closest match for each OTU. (DOCX) [file pone.0059342.s004.docx]

Table S2 –

| Sample | OTU | Closest Relative | Phylum |
| --- | --- | --- | --- |
| CE1 | CE1_1 | Bradyrhizobium sp. Pplu1-1; AF384139 | Proteobacteria |
|  | CE1_2 | uncultured soil bacterium; DS-24; AY289369 | Acidobacteria |
|  | CE1_3 | uncultured bacterium; BS125; AB240266 | Verrucomicrobia |
|  | CE1_4 | uncultured bacterium; F2_121X; GQ262999 | Chloroflexi |
|  | CE1_5 | uncultured Acidobacteria bacterium; JG35-U4-KF38; AM292625 | Acidobacteria |
|  | CE1_6 | uncultured delta proteobacterium; BuhD-66; FM877540 | Proteobacteria |
|  | CE1_7 | uncultured Acidobacteria bacterium; KBS_T1_R1_149276_a8; HM062343 | Acidobacteria |
|  | CE1_8 | uncultured bacterium; AB-Mi31; DQ347867 | Actinobacteria |
|  | CE1_9 | uncultured bacterium; FCPT428; EF516879 | Gemmatimonadetes |
|  | CE1_10 | uncultured bacterium; GBO5226bO1; HM445253 | Unclassified Bacteria |
|  | CE1_11 | uncultured Acidobacteria bacterium; HEW_08_666; HQ598734 | Acidobacteria |
|  | CE1_12 | uncultured bacterium; Elev_16S_1645; EF020189 | Acidobacteria |
|  |  |  |  |
| CE2 | CE2_1 | uncultured Acidobacteria bacterium; AMIB4; AM935448 | Acidobacteria |
|  | CE2_2 | uncultured Chloroflexi bacterium; A248; JF833502 | Chloroflexi |
|  | CE2_3 | uncultured bacterium; FFCH11914; EU132547 | Actinobacteria |
|  | CE2_4 | Bradyrhizobium sp. BR3287; AY649442 | Proteobacteria |
|  | CE2_5 | uncultured bacterium; FFCH10600; EU132327 | Acidobacteria |
|  | CE2_6 | uncultured bacterium; FCPT428; EF516879 | Gemmatimonadetes |
|  | CE2_7 | uncultured Acidobacteriales bacterium; Plot29-2E02; EU202770 | Acidobacteria |
|  | CE2_8 | uncultured bacterium; ncd2134e02c1; JF180381 | Proteobacteria |
|  | CE2_9 | uncultured bacterium; ncd138h07c1; HM259388 | Actinobacteria |
|  | CE2_10 | uncultured bacterium; FFCH5238; EU134821 | Gemmatimonadetes |
|  | CE2_11 | Bacillus sp. SD521; AB055096 | Firmicutes |
|  |  |  |  |
| CE3 | CE3_1 | uncultured bacterium; FFCH16191; EU132927 | Actinobacteria |
|  | CE3_2 | uncultured bacterium; Elev_16S_593; EF019369 | Unclassified Bacteria |
|  | CE3_3 | uncultured Gemmatimonadales bacterium; Plot29-2G07; EU202840 | Gemmatimonadetes |
|  | CE3_4 | Bradyrhizobium elkanii; STB238; HQ533242 | Proteobacteria |
|  | CE3_5 | uncultured Verrucomicrobium sp.; D.an-34; JX505101 | Verrucomicrobia |
|  | CE3_6 | uncultured bacterium; FFCH3116; EU134316 | Proteobacteria |
|  | CE3_7 | bacterium Ellin5249; AY234600 | Actinobacteria |
|  | CE3_8 | uncultured bacterium; FFCH15030; EU135332 | Unclassified Bacteria |
|  | CE3_9 | uncultured Acidobacterium sp.; BuhC-173; FM866297 | Acidobacteria |
|  | CE3_10 | uncultured Acidobacteria bacterium; HEW_08_181; HQ598492 | Acidobacteria |
|  | CE3_11 | uncultured Acidobacteria bacterium; a1; JQ825168 | Acidobacteria |
|  | CE3_12 | uncultured bacterium; GZ24; JX133440 | Proteobacteria |
|  | CE3_13 | uncultured bacterium; NR.1.076; EF494343 | Verrucomicrobia |
|  | CE3_14 | uncultured bacterium; FFCH5238; EU134821 | Gemmatimonadetes |
|  |  |  |  |
| GC1 | GC1_1 | Chelatococcus asaccharovorans; CP141b; AJ871433 | Proteobacteria |
|  | GC1_2 | uncultured bacterium; FFCH4190; EU135325 | Unclassified Bacteria |
|  | GC1_3 | uncultured bacterium; FFCH5238; EU134821 | Gemmatimonadetes |
|  | GC1_4 | uncultured Gemmatimonadales bacterium; Plot29-2G07; EU202840 | Gemmatimonadetes |
|  | GC1_5 | uncultured bacterium; P924; GQ214115 | Acidobacteria |
|  | GC1_6 | uncultured Gemmatimonadetes bacterium; Sliv-73; FM877674 | Gemmatimonadetes |
|  | GC1_7 | uncultured bacterium; TP-SL-B-71; HQ864108 | Actinobacteria |
|  | GC1_8 | uncultured soil bacterium; ABS-13; AY289452 | Proteobacteria |
|  | GC1_9 | uncultured bacterium; nbw995e03c1; GQ046291 | Actinobacteria |
|  | GC1_10 | uncultured bacterium; p7i12ok; FJ478583 | Chloroflexi |
|  | GC1_11 | uncultured bacterium; pLW-78; DQ067019 | Gemmatimonadetes |
|  | GC1_12 | uncultured bacterium; FFCH10288; EU134836 | Gemmatimonadetes |
|  |  |  |  |
| GC2 | GC2_1 | uncultured Acidobacteriales bacterium; Plot03-B06; EU276494 | Acidobacteria |
|  | GC2_2 | uncultured bacterium; FCPU547; EF515944 | Acidobacteria |
|  | GC2_3 | uncultured bacterium; F2_121X; GQ262999 | Chloroflexi |
|  | GC2_4 | uncultured Acidobacteriales bacterium; Plot29-2E02; EU202770 | Acidobacteria |
|  | GC2_5 | uncultured bacterium; FFCH10275; EU132612 | Actinobacteria |
|  | GC2_6 | uncultured Gemmatimonadales bacterium; Plot29-H02; EU202844 | Gemmatimonadetes |
|  | GC2_7 | Arthrobacter chlorophenolicus; 19; JQ958834 | Actinobacteria |
|  | GC2_8 | uncultured bacterium; FFCH5238; EU134821 | Gemmatimonadetes |
|  | GC2_9 | uncultured Acidobacteria bacterium; SEG_08_634; HQ729855 | Acidobacteria |
|  | GC2_10 | uncultured bacterium; p7c14ok; FJ479422 | Unclassified Bacteria |
|  |  |  |  |
| GC3 | GC3_1 | uncultured Acidobacteria bacterium; AKYH1017; AY922089 | Acidobacteria |
|  | GC3_9 | uncultured Gemmatimonadetes bacterium; Amb_16S_479; EF018145 | Gemmatimonadetes |
|  | GC3_2 | uncultured bacterium; ncd251e07c1; HM263028 | Unclassified Bacteria |
|  | GC3_3 | uncultured Acidobacteriales bacterium; DGGE gel band Plot21-2C12; EU192965 | Acidobacteria |
|  | GC3_4 | uncultured Acidobacteria bacterium; AEG_08_163; HQ597143 | Acidobacteria |
|  | GC3_5 | uncultured bacterium; FFCH11679; EU132546 | Actinobacteria |
|  | GC3_6 | uncultured bacterium; FFCH7024; EU134405 | Proteobacteria |
|  | GC3_7 | uncultured bacterium; FFCH5238; EU134821 | Gemmatimonadetes |
|  | GC3_8 | uncultured bacterium; FFCH15030; EU135332 | Unclassified Bacteria |
|  | GC3_10 | Methylobacterium sp. WSM3674; DQ838528 | Proteobacteria |
|  |  |  |  |
| BC1 | BC1_1 | uncultured bacterium; 1700-7; AY425767 | Proteobacteria |
|  | BC1_2 | uncultured Gemmatimonadetes bacterium; F05_WMSP2; DQ450800 | Gemmatimonadetes |
|  | BC1_3 | uncultured bacterium Riz6E2 (T); AJ244309 | Verrucomicrobia |
|  | BC1_4 | uncultured Acidobacteriaceae bacterium; CM3B03; AM936237 | Acidobacteria |
|  | BC1_5 | uncultured bacterium; Michigan 46; HQ844554 | Unclassified Bacteria |
|  | BC1_6 | uncultured Acidobacteria bacterium; JG35-U4-KF38; AM292625 | Acidobacteria |
|  | BC1_7 | uncultured Verrucomicrobia subdivision 3 bacterium; EB1106; AY395425 | Verrucomicrobia |
|  | BC1_8 | uncultured Acidobacteriales bacterium; Plot29-2E02; EU202770 | Acidobacteria |
|  |  |  |  |
| BC2 | BC2_1 | Bradyrhizobium sp. RITF322; JQ796660 | Proteobacteria |
|  | BC2_2 | uncultured organism; ctg_CGOGA56; DQ395987 | Gemmatimonadetes |
|  | BC2_3 | uncultured Acidobacteria bacterium; JG35-U4-KF38; AM292625 | Acidobacteria |
|  | BC2_4 | uncultured bacterium; TSBAR001_G06; AB486119 | Acidobacteria |
|  | BC2_5 | uncultured bacterium; FFCH10600; EU132327 | Acidobacteria |
|  | BC2_6 | uncultured bacterium; ncd242b12c1; HM269044 | Verrucomicrobia |
|  | BC2_7 | uncultured bacterium; FFCH1285; EU132786 | Actinobacteria |
|  | BC2_8 | uncultured Acidobacteria bacterium; AKYG1078; AY921950 | Acidobacteria |
|  | BC2_9 | uncultured bacterium; FCPT428; EF516879 | Gemmatimonadetes |
|  | BC2_10 | uncultured soil bacterium; 960-2; AF423301 | Actinobacteria |
|  |  |  |  |
| BC3 | BC3_1 | uncultured organism; ctg_CGOGA56; DQ395987 | Gemmatimonadetes |
|  | BC3_2 | Bradyrhizobium elkanii; JNFb1; GQ181041 | Proteobacteria |
|  | BC3_3 | uncultured Xiphinematobacteriaceae bacterium; EB1007; AY395326 | Verrucomicrobia |
|  | BC3_4 | actinomycete L5; AY534920 | Actinobacteria |
|  | BC3_5 | bacterium Ellin5082; AY234499 | Actinobacteria |
|  | BC3_6 | Arthrobacter oxydans; K22-04; EU333869 | Actinobacteria |
|  | BC3_7 | uncultured Myxococcales bacterium; Plot22-C05; EU665111 | Proteobacteria |
|  | BC3_8 | uncultured bacterium; FCPN681; EF516793 | Verrucomicrobia |
|  | BC3_9 | uncultured Gemmatimonadales bacterium; Plot29-2G07; EU202840 | Gemmatimonadetes |
|  | BC3_10 | uncultured bacterium; Luq_GN460_022; HQ445648 | Unclassified Bacteria |
|  | BC3_11 | bacterium Ellin504; AY960767 | Actinobacteria |
|  | BC3_12 | uncultured Rubrobacteridae bacterium; EB1089; AY395408 | Actinobacteria |
|  | BC3_13 | uncultured Acidobacteriales bacterium; Plot17-F04; EU440628 | Acidobacteria |
|  | BC3_14 | uncultured bacterium; WB29; JX133620 | Actinobacteria |
|  | BC3_15 | uncultured bacterium; C16-TACK-50; HE818683 | Proteobacteria |
|  |  |  |  |

CE – Cerrado / GC – Green Cane / BC – Burnt Cane.
